# GTP Cyclohydrolase I Gene Polymorphisms Are Associated with Endothelial Dysfunction and Oxidative Stress in Patients with Type 2 Diabetes Mellitus

**DOI:** 10.1371/journal.pone.0108587

**Published:** 2014-11-04

**Authors:** Pawel P. Wolkow, Wladyslaw Kosiniak-Kamysz, Grzegorz Osmenda, Grzegorz Wilk, Beata Bujak-Gizycka, Adam Ignacak, Mihir Kanitkar, Malgorzata Walus-Miarka, David G. Harrison, Ryszard Korbut, Maciej T. Malecki, Tomasz J. Guzik

**Affiliations:** 1 Centre for Medical Genomics OMICRON, Jagiellonian University Medical College, Krakow, Poland; 2 Department of Pharmacology, Jagiellonian University Medical College, Krakow, Poland; 3 Department of Internal and Agricultural Medicine, Jagiellonian University Medical College, Krakow, Poland; 4 Department of Clinical Pharmacology, Vanderbilt University, Nashville, TN, United States of America; 5 Department of Metabolic Diseases, Jagiellonian University Medical College, Krakow, Poland; 6 University Hospital, Krakow, Poland; 7 Institute of Cardiovascular and Medical Sciences, University of Glasgow, Glasgow, United Kingdom; Children's Hospital Boston/Harvard Medical School, United States of America

## Abstract

**Background:**

The genetic background of atherosclerosis in type 2 diabetes mellitus (T2DM) is complex and poorly understood. Studying genetic components of intermediate phenotypes, such as endothelial dysfunction and oxidative stress, may aid in identifying novel genetic components for atherosclerosis in diabetic patients.

**Methods:**

Five polymorphisms forming two haplotype blocks within the GTP cyclohydrolase 1 gene, encoding a rate limiting enzyme in tetrahydrobiopterin synthesis, were studied in the context of flow and nitroglycerin mediated dilation (FMD and NMD), intima-media thickness (IMT), and plasma concentrations of von Willebrand factor (vWF) and malondialdehyde (MDA).

**Results:**

Rs841 was associated with FMD (p = 0.01), while polymorphisms Rs10483639, Rs841, Rs3783641 (which form a single haplotype) were associated with both MDA (p = 0.012, p = 0.0015 and p = 0.003, respectively) and vWF concentrations (p = 0.016, p = 0.03 and p = 0.045, respectively). In addition, polymorphism Rs8007267 was also associated with MDA (p = 0.006). Haplotype analysis confirmed the association of both haplotypes with studied variables.

**Conclusions:**

Genetic variation of the GCH1 gene is associated with endothelial dysfunction and oxidative stress in T2DM patients.

## Introduction

Atherosclerosis and its clinical manifestations, such as myocardial infarction and stroke, are the major causes of mortality in type 2 diabetes mellitus (T2DM) patients [1]. While major clinical and biochemical risk factors are well known, several large randomized clinical trials show ineffectiveness of intensive therapies in the prevention of cardiovascular mortality in T2DM [2]–[4], suggesting that not all therapeutic options have already been exploited. This may be related to the complexity of the pathogenesis of atherosclerosis, which is initiated and modified by numerous genetic and environmental risk factors, many of which remain unknown. Use of quantitative phenotypes, rather than dichotomized presence of atherosclerosis or its clinical complications as outcome variables, may increase the power to detect significant genetic associations. In addition, intermediate quantitative phenotypes are usually associated with a single predominant pathomechanism of atherosclerosis [5]. Thus, studies involving such phenotypes are less prone to confounding by concomitant factors [6]. The most important mechanisms of atherosclerosis include lipid accumulation in the arterial wall [7], endothelial dysfunction [8], imbalance in redox homeostasis [9] and chronic inflammation [10]. While all of these mechanisms are important, endothelial dysfunction seems to precede the development of atherosclerotic plaque both locally [11] and systemically [12]. More importantly, endothelial dysfunction in human vessels from diabetic subjects is primarily caused by increased oxidative stress, which results primarily from the uncoupling of endothelial nitric oxide synthase (eNOS) [13]. In T2DM patients, uncoupled eNOS contributes to net superoxide excess, rather than producing protective nitric oxide (NO). This phenomenon is caused by the loss of bioavailability of the eNOS co-factor - tetrahydrobiopterin (BH_4_) and can be reversed by BH_4_ supplementation [13]. The mechanism for decreased bioavailability of BH_4_ in T2DM is not known. Several recent studies have shown that BH_4_ levels may be modified by genetic variability of the GTP-cyclohydrolase 1 (GCH1) gene located on chromosome 14 in humans and encoding a rate limiting enzyme in the complex process of tetrahydrobiopterin synthesis [14]–[16]. In the present study, we searched for the association between the polymorphisms within the GCH1 gene and selected intermediate, quantitative phenotypes related to atherosclerosis in T2DM patients. These included endothelial dysfunction (flow mediated dilation – FMD) and its biochemical parameter (von Willebrand factor – vWF), measures of oxidative stress (malondialdehyde levels – MDA), and subclinical atherosclerosis (intima-media thickness – IMT).

## Materials and Methods

### Study population

The study population consisted of 182 consecutive patients diagnosed with T2DM and followed up in an outpatient setting at the Department of Metabolic Diseases, University Hospital, Krakow, Poland. During patient recruitment, the WHO definitions and criteria of diabetes diagnosis were used [17]. All patients were white Caucasians and inhabitants of southeastern Poland. Upon examination, demographic data and information about the course of their disease, family history, past and current treatments and lifestyle habits were obtained. Only patients with clinical diagnosis of T2DM and no insulin therapy, within the first year of initial diagnosis, were included in this study. Blood for DNA isolation and biochemical measurements, including MDA and vWF plasma concentrations, was obtained by antecubital venipuncture. Vasodilation and IMT were measured ultrasonographically with an 8 MHz probe by a single researcher, blinded to other data. Diagnosis of diabetic complications and biochemical measurements were performed as previously described [17]. The study was approved by the Bioethics Committee of the Jagiellonian University. All patients provided informed consent.

### Assessment of endothelial function

Endothelial function was assessed by flow mediated dilation (FMD) measurement as described and validated by our laboratory before [17], [18]. Measurements were performed with the Toshiba Xario Diagnostic Ultrasound System and an 8MHz linear transducer. Briefly, measurements were recorded before 5 minute-long brachial artery occlusion and then subsequently 1, 2 and 5 minutes after cuff deflation. Measurements during diastole were recorded and maximal dilation (usually detected within ca. 60–120 sec) was analyzed and reported. Maximal nitroglycerine mediated dilation (NMD) was measured to study non-endothelium dependent vasodilations. vWF concentration in plasma was analyzed using a commercial ELISA kit from DAKO (Glostrup, Denmark).

### Measurement of IMT

Measurement of IMT was performed as previously described [17], [18] at 12 different points along the right and left common carotid artery. Mean (IMTmean) and maximal IMT (IMTmax) were calculated.

### MDA determination

MDA in plasma was determined using liquid chromatography with mass detection (HPLC/MS, LCQ Finnigan Matt). These tests were performed at the Department of Pharmacology JUMC by a modified method described by Sim et al. [19]. Plasma samples were incubated with NaOH to liberate bound MDA and perchloric acid to precipitate proteins. The supernatant was then subjected to extraction twice with n-hexane. The separated organic phase was analyzed by HPLC/MS.

### Genotyping

**Figure 1:**
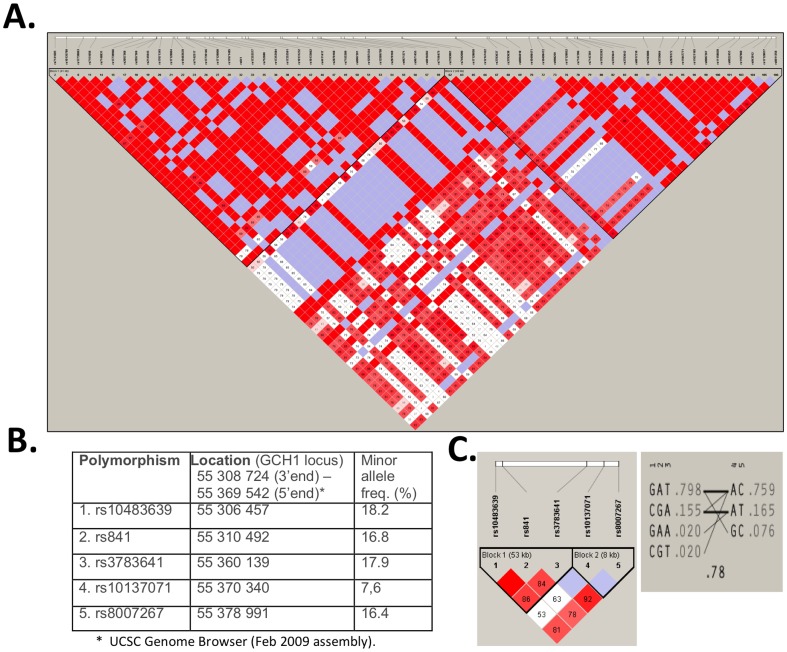
LD within GCH1 gene locus. (A) Extent of LD between polymorphisms at the GCH1 gene locus, based on HapMap Phase II data. Two black triangles designate two haplotype blocks of tight LD. (B) Physical location and minor allele frequency of 5 genotyped polymorphisms from GCH1 locus according to UCSC Genome Browser (Feb 2009 assembly). (C) LD analysis between 5 polymorphisms, genotyped in T2DM subjects from the Polish population, forms two separate haplotype blocks. Data were analyzed and visualized with Haploview ver. 4.2. Haplotype blocks were defined based on the solid spine of LD rule. Numeric values represent Lewontin's D′ between pairs of polymorphisms.

Polymorphisms in the GCH1 gene were selected for genotyping based on the existing literature [20], [21]. The GCH1 gene spans slightly over 60 kb on human chromosome 14 (physical location 54,378,474–54,439,292 on March 2006 build of human genome according to UCSC genome browser). Linkage disequilibrium (LD) in a European (Tuscany, Italy) and European descent (Utah, USA) populations (CEU + TSI) from HapMap data [22] is shown in Figure 1A. These populations combined are probably the best available approximation of LD extent for White Caucasians, such as a Polish population from Central Europe. Data show the existence of two haplotype blocks of very tight LD, extending beyond the gene boundaries. The boundary between these two blocks is localized in the second intron, dividing the gene into two thirds in the 5′-end block and one third in the 3′-end block. Therefore, the polymorphisms for this association study were selected from both haplotype blocks (Figure 1B). DNA was isolated from peripheral blood leukocytes using a guanidinium thiocyanate method (DNAzol, Life Technologies).

Polymorphisms rs10483639, rs841, rs3783641, rs10137071, and rs8007267 were determined using commercially available kits ABI TaqMan SNP Genotyping pre-designed assays (Applied Biosystems, USA). End-point fluorescence was determined using Applied Biosystems 7900HT Fast Real-Time PCR System (Applied Biosystems, USA).

### Statistical analysis

Data for statistical analysis were analyzed with SAS ver. 9.3 (Cary, USA). Deviations of genotype frequencies of studied polymorphisms from Hardy-Weinberg equilibrium were tested using a modified Chi-square test, implemented in the SAS Genetics module. An extent of LD between the studied polymorphisms was calculated and presented graphically with Haploview ver. 4.1. Patients with different genotypes were compared for differences in continuous variables using the Wilcoxon test and in categorical variables using Chi-square or Fisher's exact test. To compare the effect of polymorphisms and haplotype blocks on quantitative outcome variables, we used PLINK ver. 1.06 with association tests based on Wald statistic. Univariate tests of association were used for individual polymorphisms and haplotype blocks, multivariate tests for combinations of polymorphisms and clinical variables. The following clinical variables, potential confounders of association between genetic factors and outcome variables, were tested: age and sex of patients, characteristics of T2DM, risk factors of atherosclerosis and medication used. P values of less than 0.05 were considered statistically significant.

## Results

### Study population

Clinical characteristics of patients are summarized in Table 1. These data include demographic characteristics (sex and age), major risk factors for atherosclerosis, current therapies, history of diabetes and its complications, cardiovascular diseases, lipid profile, and outcome phenotypes (FMD, NMD, vWF, and MDA).

**Table 1:** Clinical characteristics of the studied T2DM patients.

Clinical feature	Value in 182 patients *
Female sex (%)	50
Age at examination (years)	56.5 [37–72]
**Characteristics of T2DM**	
Use of oral antidiabetic drugs (%)	63.2 **
Use of insulin (%)	52.8 **
Any form of retinopathy (%)	39
Nephropathy (%)	16.5
Neuropathy (%)	34.1
Age at diagnosis (years)	47 [26–68]
Duration of diabetes (years)	9 [1]–[31]
Current blood glucose (mmol/L)	9.2 [4.3–34]
Hemoglobin A1c (%)	7.6 [5.1–14.2]
Peptide C (ng/mL)	3 [0.15–11.5]
Serum creatinine (µmol/L)	75.3 [47–386.4]
Renal dysfunction; serum creatinine>155 µmol/L (%)	5.0
ALAT (IU/L)	20 [8–90]
**Risk factors for atherosclerosis**	
Hypertension (%)	88.5
Ever smoking status (%)	59.1
Current smoking status (%)	21.6
Overweight/Obesity (%)	Overweight –29.7; Obese –59.9
BMI (kg/m^2^)	31.4 [21–56.4]
Total cholesterol (mmol/L)	5.1 [2.7–19.6]
LDL cholesterol (mmol/L)	2.9 [0.5–7.3]
HDL cholesterol (mmol/L)	1.1 [0.4–2.5]
Triglycerides (mmol/L)	1.9 [0.6–14.1]
**Atherosclerosis and CAD**	
Ischaemic heart disease (%)	47.8
CCS classification (%)	0–50; 1–24.7; 2–17.6; 3–7.7
PTCA performed in the past (%)	11.5
Myocardial infarction in the past (%)	12.1
Presence of atherosclerotic plaque (%)	60.4
**Medications**	
ACE inhibitors (%)	76.9
diuretics (%)	50
beta – blockers (%)	37
calcium blockers (%)	23.6
alpha – blockers (%)	7.7
nitrates (%)	14.8
aspirin (%)	43.4
statins (%)	49.7
**Studied vascular endpoints**	
IMT (mm, average of 12 measurements)	0.84 [0.55–1.1]
IMT (mm, maximum of 12 measurements)	1 [0.7–1.5]
FMD (ratio to baseline at 5 minutes)	1.03 [1–1.24]
NMD (ratio to baseline at 5 minutes)	1.12 [1–1.31]
MDA (µmol/L)	3.32 [0.16–22.3]
vWF (% of normal population)	101.3 [37.5–144.1]

* Frequency of occurrence among patients for categorical variables or median [minimum; maximum] value for continuous variables.

** At the time of examination 42 T2DM patients use both insulin and oral hypoglycemic drugs, 54 use only insulin, 73 use only oral hypoglycemic drugs, 13 use neither insulin nor oral hypoglycemic drugs (diet and physical exercises only).

### LD in GCH1 gene in T2DM patients in Poland

The physical location of 5 genotyped polymorphisms on chromosome 14 is described in Figure 1B. Linkage analysis of genotyping data obtained in our study population revealed the existence of 2 haplotype blocks, similar to the results obtained using analysis of HapMap data (Figure 1A–C). Polymorphisms rs10483639, rs841, and rs3783641 belong to the first haplotype block while polymorphisms rs10137071 and rs8007267 belong to the second haplotype block.

### GCH1 genetic polymorphisms and vascular outcome variables

**Figure 2:**
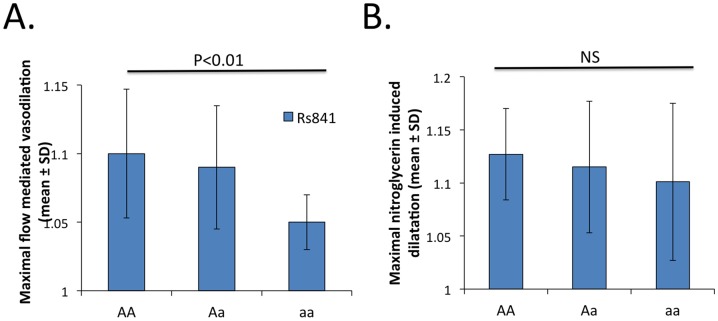
Relationship between rs841 polymorphism in the 3'-UTR of GCH1 gene and vascular function measured as endothelium dependent FMD (panel A) and endothelium independent NMD) (panel B). AA indicates frequent homozygote, Aa - heterozygote, aa - rare homozygote.

**Figure 3:**
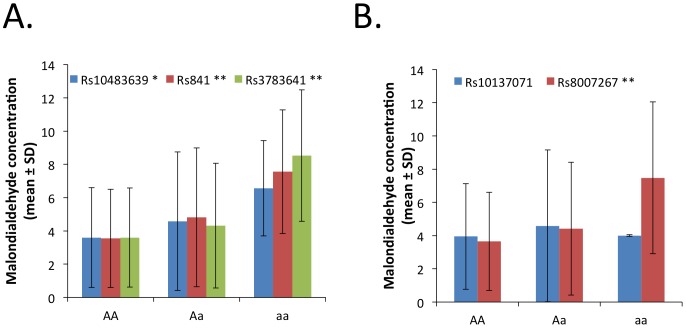
Relationship between studied polymorphisms and plasma vWF concentration. Relationship to polymorphisms forming haplotype block 1 (panel A) and haplotype block 2 (panel B) is demonstrated. Statistical significance of linear regression: * p<0.05.

**Figure 4:**
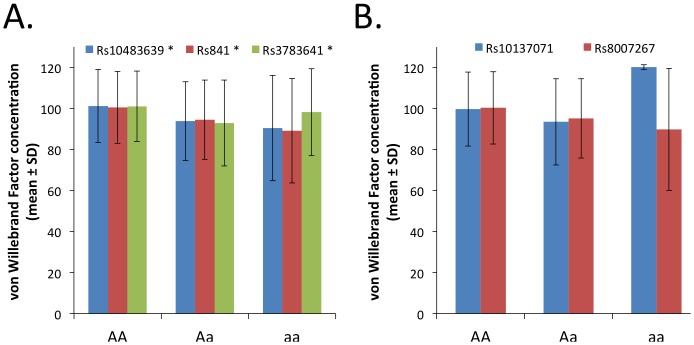
Relationship between studied polymorphisms and plasma MDA concentration measured by mass-spec analysis. Relationship to polymorphisms forming haplotype block 1 (panel A) and haplotype block 2 (panel B) is demonstrated. Statistical significance of linear regression: * p<0.05; ** p<0.01.

We next studied the associations between each of the 5 genotyped SNPs, as well as each of the 2 haplotype blocks formed by these SNPs, with outcome variables (Table 2). Polymorphism rs841 was associated with endothelial function measured as maximal FMD (Figure 2A), while no association was found with control NMD (Figure 2B). Three polymorphisms belonging to the same haplotype block (rs10483639, rs841, and rs3783641) were associated with vWF concentration, a plasma biomarker of endothelial function - (Figure 3A and B). In the case of the last polymorphism, rs3783641, for the genotype mean value of vWF concentration we observed no progression from Aa to aa genotypes. This may suggest either a non-additive model of association or simply reflect the fact that there were only 6 people with the aa genotype in our study. Interestingly, 4 polymorphisms (rs10483639, rs841, rs3783641, and rs8007267) were also associated with plasma MDA concentration, a systemic oxidative stress marker (Figure 4A and B).

**Table 2:** Association of individual GCH1 gene polymorphisms with outcome variables.

Outcome variable	SNP	P value
FMD (max)	Rs10483639	0.056
	Rs841	**0.01**
	Rs3783641	0.09
	Rs10137071	0.63
	Rs8007267	0.47
NMD (max)	Rs10483639	0.24
	Rs841	0.25
	Rs3783641	0.19
	Rs10137071	0.29
	Rs8007267	0.07
IMT (mean)	Rs10483639	0.15
	Rs841	0.22
	Rs3783641	0.08
	Rs10137071	0.28
	Rs8007267	0.11
IMT (max)	Rs10483639	0.21
	Rs841	0.09
	Rs3783641	0.06
	Rs10137071	0.40
	Rs8007267	0.13
MDA	Rs10483639	**0.012**
	Rs841	**0.0015**
	Rs3783641	**0.003**
	Rs10137071	0.51
	Rs8007267	**0.006**
vWF	Rs10483639	**0.016**
	Rs841	**0.03**
	Rs3783641	**0.045**
	Rs10137071	0.75
	Rs8007267	0.055

### Association of haplotypes with outcome variables

Association of each of the two described above haplotype blocks with studied outcome variables was determined using omnibus haplotype association tests with studied variables (Table 3).

**Table 3:** Association of two GCH1 gene haplotype blocks, formed by 5 studied polymorphisms with outcome variables.

Studied variable	Haplotype block *	P value for association
FMD max	1	0.27
	2	0.72
NMD	1	0.6
	2	0.16
IMT (average)	1	0.5
	2	0.1
IMT (max)	1	0.44
	2	0.16
MDA	1	**0.0253**
	2	**0.0145**
vWF	1	**0.00285**
	2	0.12

* Haplotype block 1 consists of the following SNPs: Rs10483639, Rs841, Rs3783641 (estimated haplotype frequencies: GAT 79.9%; CGA 15.4%; GAA 2%; CGT 2%. Haplotype block 2 consists of the following SNPs: Rs10137071, Rs8007267 (estimated haplotype frequencies: AC 75.5%; AT 16.8%; GC 7.7%).

Haplotype analysis showed that MDA concentration was associated with both haplotype blocks while vWF concentration was associated only with the first haplotype block. This is in line with the analysis of associations of individual polymorphisms with outcome variables. In addition, we observed that association of both haplotype blocks with MDA concentration was less significant than the ones of individual polymorphisms. However, in case the of vWF concentration, the association of the first haplotype block was more significant than that of any individual polymorphism. There was no significant association between the haplotype blocks and FMD values.

### Clinical variables as potential confounders of GCH1 polymorphisms associations

In addition to single polymorphism analyses and haplotype block analyses, we have also tested whether clinical covariates, described in the Statistical Analysis paragraph of the Materials and Methods section, changed the association between GCH1 polymorphisms and the outcome variables – FMD as well as MDA and vWF concentrations. The only covariate that significantly associated on its own with the outcome variables was current smoking status. However, we also forced in kidney dysfunction (defined as creatinine concentration above 155 ml/L) and duration of diabetes into the multivariate models, as these are known to particularly affect endothelial function in diabetes (Table 4). The relationships between polymorphisms and outcome variables were independent of the confounding variable of current smoking status. Changes in beta coefficients of association for any studied SNP were less than 10% upon addition of current smoking as a covariate in the model. Thus, covariates did not confound this relationship.

**Table 4:** Models of association of GCH1 gene polymorphisms with outcome variables, accounting for current smoking, kidney dysfunction* and diabetes duration covariates.

Outcome variable	Model including single SNP	Model including SNP and covariates	Beta coefficient	P value
**FMD max**	rs841		−0.018	0.01
		rs841	−0.017	0.015
		Current smoking	−0.011	0.2
		Renal dysfunction	−0.0009	0.17
		Diabetes duration	−0.0022	0.93
**MDA**	rs10483639		1.216	0.01
		rs10483639	1.042	0.03
		Current smoking	−0.84	0.15
		Renal dysfunction	0.06	0.23
		Diabetes duration	−0.8	0.42
	rs841		1.583	0.001
		rs841	−0.755	0.19
		Current smoking	0.04	0.35
		Renal dysfunction	−1.341	0.44
		Diabetes duration	1.431	0.005
	rs3783641		1.457	0.003
		rs3783641	1.298	0.01
		Current smoking	−0.931	0.10
		Renal dysfunction	0.0412	0.35
		Diabetes duration	−1.068	0.50
	rs8007267		1.337	0.006
		rs8007267	1.136	0.024
		Current smoking	−0.862	0.14
		Renal dysfunction	0.05	0.28
		Diabetes duration	−1.243	0.44
**vWF**	rs10483639		−6.453	0.016
		rs10483639	−5.706	0.033
		Current smoking	−4.99	0.12
		Renal dysfunction	0.098	0.69
		Diabetes duration	16.91	0.03
	rs841		−5.839	0.031
		rs841	−5.371	0.047
		Current smoking	−4.546	0.13
		Renal dysfunction	−0.069	0.78
		Diabetes duration	15.47	0.065
	rs3783641		−5.445	0.045
		rs3783641	−4.728	0.081
		Current smoking	−4.486	0.15
		Renal dysfunction	−0.0389	0.87
		Diabetes duration	16.8	0.03

* Renal dysfunction is defined as patients having creatinine concentration above 155 µmol/L.

## Discussion

In the present study we have found that polymorphisms in the GCH1 gene, that can affect tetrahydrobiopterin synthesis, are associated with markers of endothelial dysfunction and oxidative stress. This is important, as both endothelial dysfunction and oxidative stress in T2DM patients are the major mechanisms for the development of both micro- and macro-angiopathy. Further studies are required to determine if the same association of polymorphisms is true for patients with type 1 diabetes. Expanding this study to include a group of healthy, non-diabetic control subjects could also verify whether the described associations of polymorphisms with endothelial dysfunction and oxidative stress also remain significant during normoglycemia or are specific to diabetic patients only.

Functional effects of polymorphisms located in the *GCH1* gene locus have been initially shown in relation to the role of BH4 in catecholamine and serotonin synthesis [23]. Rare mutations in *GCH1* lead to hyperphenylalaninemia [24] or DOPA-responsive dystonia [25]. However, the association of sequence differences in this gene with NO biology have been quickly established. For example, the GCH1 haplotype with population frequency of 15.4% has been linked to lower pain sensitivity due to decreased NO production [15]. Although this association has not been replicated [26], recent interest has focused on the relationship with cardiovascular disease. Importantly, Lotsch et al. [27] demonstrated that the pain-protective haplotype can be inferred by genotyping just three polymorphisms, which we have included in our analysis.

A number of studies have looked at cardiovascular disease in relation to the GCH1 genetic variation. Zhang et al. [20] reported that a GCH1 polymorphism in the 3′-UTR is associated with cardiovascular risk, due to decreased NO production and higher blood pressure in carriers of one of the alleles. Subsequently, Doehring et al. [21] showed that this 3′-UTR polymorphism is in complete LD with the pain-modulating haplotype. Polymorphisms rs1049255 and rs841 were independently associated with coronary artery disease [28]. According to this study, epistatic interactions with other polymorphisms of the NO biosynthesis pathway contribute additionally to the risk of coronary artery disease. The first direct link of genetic variation with BH4 levels was described by Antoniades et al. [16]. They observed lower BH4 levels in carriers of the deleterious GCH1 haplotype, which results in increased production of vascular superoxide and decreased acetylcholine mediated vasodilation in saphenous vein fragments. Considering this evidence and the role BH4 plays in diabetic vascular disease, the importance of genetic variation of the GCH1 gene in T2DM patients is particularly interesting. Polymorphism rs841, which forms a part of the first haplotype block, has been implicated in macrovascular disease in the Chinese cohort of T2DM patients [29]. In this earlier study, carriers of the deleterious genotype were characterized by lower plasma NOx concentrations and lower FMD but higher MDA level and IMT.

In the present project, we have studied 5 polymorphisms spread throughout the gene, which has allowed us to look at variation in the GCH1 gene. Careful examination of the structure of LD in the Polish cohort of T2DM patients showed that these polymorphisms belong to two separate haplotype blocks, in agreement with our analysis of HapMap data. A previous study reported the existence of various numbers of haplotype blocks in the GCH1 locus, depending on the population studied [15]. Two earlier papers suggested tight LD across the whole GCH1 locus [16], [21]. However, Zhang and et al. [20], who performed a resequencing study, report two distinct haplotype blocks in subjects from Sub-Saharan Africa and a lack of LD of the 3′-end polymorphism with other areas of the gene in subjects of European ancestry. Therefore, our careful examination of the structure of LD in the Polish population in the context of HapMap data is of great interest.

We found that only one of the polymorphisms, rs841, was associated with impaired endothelium dependent vascular dilatation while no relationship was observed with non-endothelium dependent responses. This is in agreement with previous reports of the functional effects of this polymorphism on vascular function in T2DM. Subsequently, we found that three polymorphisms, which form the first haplotype block, were also associated with biomarker plasma vWF concentration, which clearly confirms the functional importance of the GCH1 gene variability in the regulation of vascular function in T2DM.

The major mechanism through which the GCH1 gene could affect endothelial function is eNOS uncoupling, which is directly linked to oxidative stress. Four polymorphisms, three constituting the first haplotype block and one from the second haplotype block, are associated with MDA plasma concentration. Moreover, the first haplotype block was even more strongly associated with this variable than any of the analyzed SNPs. This finding suggests that the first haplotype block may contain a non-genotyped polymorphism affecting MDA concentration. Therefore, this would be a stronger association than any of the polymorphisms previously genotyped by our group. An alternative explanation to this finding could be an epigenetic interaction between studied polymorphisms or other polymorphisms in haplotype block 1, modifying the presence of CpG islands in this locus, thereby affecting methylation pattern and expression of GCH1. Such a possibility was demonstrated at other loci [30]. In the case of vWF, association of the first haplotype block with this variable was less significant than with individual SNPs.

We also tested whether associations defined in this study could have been confounded by clinical factors. The only clinical variable associated with the outcome variables of MDA and vWF concentrations (but not with FMD), was the current smoking status. However, further statistical analysis has shown that the association of SNPs with outcome variables was not confounded by smoking, renal dysfunction, or diabetes duration. In summary, GCH1 polymorphisms or haplotype blocks are associated with FMD, malondialdehyde, and von Willebrand factor concentrations, independently of other clinical characteristics in Polish T2DM patients.

Interestingly, in our T2DM study group we did not observe an association between GCH1 genetic variation and IMT, which has been reported previously [29]. It is possible that our patients were younger and their IMT was overall not substantially increased (mean IMT below 0.9 mm). This could have implicated that we studied a population of T2DM patients at early stage of the development of atherosclerotic disease in whom an increase of IMT not yet developed. However, as discussed above, our data show that the functional polymorphisms, which may affect NO production, can also influence intermediate phenotypes of endothelial dysfunction and imbalanced redox homeostasis [31]. We cannot exclude an alternative explanation that slight interindividual differences in IMT, measured in fraction of millimeters are harder to associate with effects of polymorphism. Such a relationship would be easier to find in a larger population.

Macrovascular complications are responsible for mortality excess and lower quality of life in T2DM patients. Their occurrence may be accelerated and exacerbated by a deficiency in NO production [32]. While NO donors (such as nitrates) are widely used in some clinical situations, their protective efficacy in coronary artery disease has been questioned in numerous clinical trials [33]. Therefore, they do not constitute a first line treatment any longer. Correction of BH4 deficiency might be a better therapeutic option correcting not only NO deficiency but oxidative stress as well. For example, it has been postulated that in carriers of susceptibility GCH1 gene polymorphisms with elevated MDA, BH4 may be oxidized to BH2 and uncouple NO synthase, which leads to further production of reactive oxygen species by eNOS, rather than NO itself [34]–[36]. The findings of our study together with published evidence of the functional importance in GCH1 gene variation may in future form a basis for diagnostic screening tests to stratify the risk of early development of macrovascular complications in newly diagnosed T2DM patients. This also presents potential therapeutic implications that will need to be clinically tested. An interesting question would be whether supplementation of BH4 might improve the clinical course of T2DM macrovascular complications in relation to stratification based on GCH1 gene polymorphisms. Stratification of patients' therapy according to their genotype is an important aspect of personalized medicine, which becomes even more possible with the advent of new, genome-wide laboratory tools.

Apart from the correction of NO availability, another important strategy for preventing complications in diabetic patients could be to reverse endothelial progenitor cell dysfunction [37]. GCH1was demonstrated to effectively reverse such dysfunction and promote re-endothelization, which may be important for wound healing [37], [38]. A group of patients who are particularly prone to complications due to endothelial dysfunction, and might therefore especially benefit from genotype-based BH4 correction, are patients with kidney-pancreas transplantation [39]–[41].

In summary, the results of our study indicate that functional polymorphisms of the GTP cyclohydrolase I gene, which directly affect tetrahydrobiopterin synthesis, are associated with endothelial dysfunction and oxidative stress in T2DM patients.
